# Evolution of Interfacial Shear Strength and Mean Intrinsic Single Strength in Biobased Composites from Bio-Polyethylene and Thermo-Mechanical Pulp-Corn Stover Fibers

**DOI:** 10.3390/polym12061308

**Published:** 2020-06-08

**Authors:** Quim Tarrés, Mònica Ardanuy

**Affiliations:** Departament de Ciència I Enginyeria de Materials, Universitat Politècnica de Catalunya (UPC), Colom 1, 08222 Terrassa, Spain; monica.ardanuy@upc.edu

**Keywords:** bio-polyethylene, natural fibers, composites, biobased, sustainable

## Abstract

In this article, with the aim of promoting sustainability, contributing to the circular economy and the fight against climate change, the production of composite materials from Bio-polyethylene reinforced with corn stover fibers has been studied. The behavior of the materials obtained has been studied experimentally and by mathematical models of micromechanics. The composite materials were produced by extrusion and then injection with from 10 to 50 wt.% of fibers. The creation of a good fiber-matrix interface was studied by the incorporation of coupling agent between (0–8 wt.%). Increase of 131.2% on tensile strength for 40wt.% reinforcement was achieved by adding 6 wt.% of coupling agent. The correct interface was demonstrated by a correlation of 0.99 between the experimental results and the results of the mathematical models used.

## 1. Introduction

World population growth and natural resource constraints have placed the concept of the circular economy at focus in recent years. The manufacture of products from plastic materials has spread enormously since the beginning of the last century. Today, plastic is used to make or package many of the products we buy and consume. More than 240 billion kilograms of plastic are produced worldwide every year. This increase in the use of plastic is due to the fact that it is a cheap and easy material to produce and because its useful life is long. However, as it is known, the massive use of plastics derived from fossil resources has generated a significant negative impact on the environment: the high persistence of plastics and the high consumption of non-renewable resources. Much of it ends up in the world’s oceans. Plastic does not degrade in the ocean. It’s only broken down into small particles. In the Pacific Ocean, a vast area, nearly 10 million square kilometers, is teeming with tiny pieces of oil-based plastic that can poison ocean life. During the last decades, the manufacture of composite materials reinforced with natural fibers has become a field of interest in the search for new biobased materials [1,2,3,4].

The use of natural fibers as reinforcement for thermoplastics has generated great interest due to the fact that it is possible to obtain improvements in mechanical properties as well as a reduction in the use of non-renewable materials, a reduction in the emission of polluting gases and the biodegradable character of the fibers at the end of the useful life of the material [5]. Natural fibers can be obtained from different sources. In this sense it is necessary to distinguish between the fibers coming from: (i) wood sources such as eucalyptus or pine [6,7,8]; (ii) vegetable fibers such as jute, abaca, flax or other materials [9,10]; (iii) recycled fibers such as recycled paper [11,12]; (iv) fibers from agro-forestry residues [13,14,15]. These materials can have very different properties due to their nature, resulting in composite materials with different properties. In addition, natural fibers can be obtained from the raw material using different methodologies, including mechanical, semi-chemical and chemical treatments [16,17]. Depending on the methodology applied, the chemical and morphological composition of the fiber will be different, resulting in different final material properties.

On the other hand, for decades, the scientific community has been studying the production of plastics from renewable sources and/or biodegradable with the aim of replacing plastics of fossil origin and avoid their persistence after use [18]. This study has led to the development of biodegradable plastics (-for the conservation of the environment through the degradation of the material) and bio-based plastics (those that are produced from renewable sources such as biomass-) [19]. Biodegradable plastics are used for short-lived applications such as packaging, bags or single-use materials. However, for long-life applications bio-based plastics are the best option. This is due to the difficulty of controlling the biodegradation process in different environments [20]. Currently, one of the most widely used plastics on the market is polyethylene. Although this polymer has been produced mainly from petroleum, nowadays, bio-based polyethylene (BioPE) plastics produced from renewable resources such as sugarcane are commercially available [21]. The polymer was introduced to the market on a commercial scale in 2019, and its production volume is expected to increase by a factor of six to 2024. As BioPE has the same chemical characteristics as polyethylene obtained from fossil resources, physical and mechanical behavior similar in terms of durability, water absorption, mechanical performance, among others, are expected from this bio-based plastic. One of the main benefits of BioPE is the use of bio-based raw material and the ability to sequester significant amounts of atmospheric CO_2_, captured by the sugarcane plant. Additionally, bio polyethylene can be recycled through the same processes as fossil-regulated polyethylene and can be incorporated into current production processes. For a circular economy, bio-polyethylene has the potential of a closed loop compared to fossil-based polyethylene, since the biogenic carbon absorbed by the sugar cane is released back into the atmosphere after biodegradation or incineration and can be absorbed again by a plant [22]. Nonetheless, it must be taken into account that an industrial production for high consumptions of bio-based plastics must overcome important challenges such as cost, technical feasibility, infrastructure and variability in properties [21,23]. One of the options to reduce the cost of production of materials, increase mechanical properties and increase sustainability is to combine these bio-based plastics with natural fibers in the form of composites [24,25]. In addition, it is possible to obtain a totally bio-based and recyclable material that can replace conventional synthetic compounds.

Corn, like rice, is one of the largest agricultural crops since is one of the basic foods in many diets worldwide. Its production reached 1038 million tons during 2014. Its cultivation generates lignocellulosic wastes from the stalk, leaves and husk. This biomass is available and rarely recovered for upcycling applications. In most cases, this agricultural by-product is burned in situ, causing the emission of polluting gases. The use of this corn stover for the production of composite materials, would allow to eliminate this source of contamination, at the same time, that to endow to this agricultural byproduct of value for the industry. By means of a thermo-mechanical digestion process (TMP) it is possible to obtain high yield lignocellulosic fibers for further processing [26,27].

It is widely known that one of the main aspects to be taken into account in the manufacture of composite materials is the interaction between the polymer matrix and the reinforcement. In the case of short fiber reinforced semi-aligned composites, the mechanical properties are mainly affected by the intrinsic properties of the natural fibers, the fiber content, the orientation of the fibers within the matrix and the stiffness of the matrix. As well as the interfacial adhesion between fiber and matrix except for the Young’s modulus of the composite. The hydrophobic nature of most of plastics as opposed to the hydrophilic nature of natural fibers represents a difficulty in this respect. Physical and chemical treatments of natural fibers can be used to avoid or reduce different and incompatible surface polarities [28,29,30]. A good fiber-matrix interaction is crucial to produce a composite with a high tensile strength. In this sense, the use of a coupling agent together with the presence of lignin in the chemical composition of the corn fibers should result in a good interface [31,32,33]. In addition, the coupling agent also helps to increase the dispersion and distribution of the fiber. For the use of natural fibers and BioPE, the use of polyethylene maleic anhydride (MAPE) allows for improved interfacial bonding. Anhydride groups form covalent bonds by esterification with hydroxyl groups of fibers and interlacing the polymer chain with the polyethylene [34,35,36].

The main objective of this work is to analyze the mechanical behavior of Bio-polyethylene composite materials reinforced with variable content of corn stover fibers (from 10 to 50 wt.%). Through the study of mechanical properties (tensile strength, Young’s modulus and deformation) and micromechanical properties (orientation factor, interfacial shear strength and intrinsic resistance of the fibers), the behavior of the materials produced will be evaluated.

## 2. Materials and Methods

**Figure 1:**
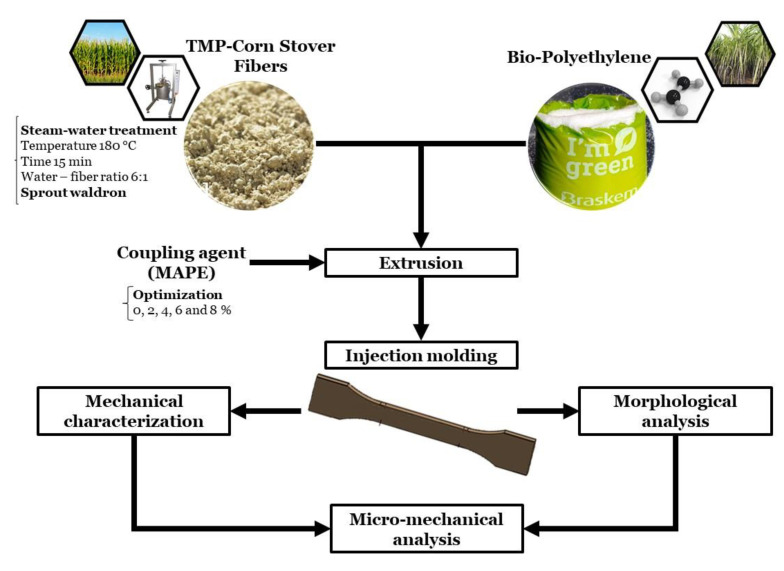
Chart flow of the present study.

Figure 1 shows the chart flow followed in the present study.

### 2.1. Materials

Bio-polyethylene injection grade SHA7260 was supplied by Braskem (Sao Paulo, Brazil) and has a molecular weight of 61.9 g/mol. Polyethylene functionalized with maleic anhydride (MAPE) Fusabond MB100D acquired from Eastman Chemical Products (San Roque, Spain) was used as coupling agent. All the reagents used in the characterization of the fibers were acquired from Scharlab Spain (Barcelona, Spain). Corn stover was supplied by Fundació Mas Badia (La Tallada d’Empordà, Spain).

### 2.2. Methods

#### 2.2.1. TMP Corn Stover Fibers’ Preparation and Characterization

Initially, the corn stover was cut by a blade mill equipped with a 5 mm sieve. The particles obtained were subjected to a thermomechanical digestion process (TMP) by means of a pressure reactor. The thermomechanical digestion process consisted of steam-water treatment at 180 °C during 15 min. The liquid ratio was kept at 6:1 (six parts of water for each part of dry weight of fibers). After the digestion, the fibers obtained were washed with abundant water and then passed through a Sprout-Waldron equipment (Andritz, Spain). This process leads to fibers with higher specific surface due to a defibrillation mechanism.

To determine the fiber morphology before and after producing the composites, the fibers were extracted from the material after the processing of the composites via injection molding. The extraction of the fibers was done with a Soxhlet apparatus using decaline as solvent for Bio-PE. The morphology of the fibers resulting from the digestion process and those extracted from the composite material was analyzed by a MORFI laboratory equipment (Techpap, France). A 25 mg/L fiber suspension has been prepared for this analysis. Five measurement repeats are performed, and 30,000 fibers are analyzed in each of them. By means of this analysis, the average length and diameter of the fibers are obtained, as well as their distribution.

The ash, extractive, lignin, cellulose and hemicellulose contents of the fibers were determined before and after the thermomechanical treatment. The ash content was determined by gravimetric analysis after calcination at 575 °C for 3 h with a Heron serie8D (Nikkelstraat, Nederland) (ASTM D1102-84 2001). For the ash determination, 2 g of sample were placed in porcelain crucibles (previously cleaned and dried) and placed in an electric muffle furnace. The initial temperature was 100 °C and the temperature was slowly raised to 525 °C to ensure the carbonization of the sample without flaming. The extracts were calculated by weight difference once extracted with 95% ethanol for 6h with a Soxhlet apparatus. The acid-soluble lignin was determined by spectrophotometric method with a Shimadzu UV/160A (Duisburg, Germany) (TAPPI UM 250 1991) and the Klason lignin by acid hydrolysis according to TAPPI T22 Om-98 1985. 2 g of each sample without extraction were placed in 100 mL beakers. Then, 40 mL of cold (10 °C) 72% sulfuric acid was added to each glass. This operation was done gradually under constant agitation and the beaker was placed in a cold water bath to mitigate the heating of the sample. Then the beaker was covered with a watch glass and kept in a bath at 20 °C for 2 h. The material was stirred frequently to ensure a complete solution. After this time, the beaker material was transferred to a flask containing 400 mL of deionized water, adding more water until a concentration of 3% sulfuric acid was achieved, and then boiled for 4 h. Additional water was added to maintain the total volume. The flask was left overnight to promote lignin precipitation and then the liquid was filtered through a 0.22 m pore size nylon membrane, passing through hot water with to remove the free acid. The acid insoluble lignin was dried on 105 °C until it remained constant by weight. The hemicellulose content was determined by high performance anion exchange chromatography (HPAEC) following the procedure described in TAPPI T223 cm-01 2001. Finally, the cellulose content was determined as the difference with the rest of the contents.

#### 2.2.2. Composites’ Production and Characterization

The TMP corn stover fibers were incorporated into the Bio-PE matrix together with the MAPE coupling agent by means of a Gelimat intensive kinetic mixer (Dusatec, New Jersey, USA). Compounding was performed for 2 min at 3000 rpm to reach melting temperature and was discharged when the temperature was around 210 °C. Different percentages of coupling agent were tested for compounds with 20 and 40 wt.% TMP corn stover fibers. With the optimized MAPE addition percentage, compounds with 10 to 50 wt.% TMP corn stover fibers were produced. The blends were pelletized and dried at 80 °C until their use for the production of samples by injection.

Standard composite specimens, dog bones with 115 mm in length of narrow grips, 13 mm of gage width and 3.2 mm of thickness, (ASTM D638-14) were produced by injection molding using an Arburg 220M 350-90U injection machine (Lossburg, Germany). The injection process was carried out with a temperature profile of 180, 190, 200 and 210 °C, using a first pressure of 120 kg·cm^−2^ and a second pressure of 37.5 kg·cm^−2^.

The physical characterization of the materials obtained consisted in the determination of the density by means of a pycnometer and the determination of the melt flow index of the material (MFI). To determine the MFI, a CEAST plastometer (Pianezza, Italy) was used. The plastometer is equipped with a capillary heated by two independent thermal resistances. The standard samples obtained were kept at 23 °C and 50% RH for 48 h in a climatic chamber before testing the mechanical properties (ASTM D618). Tensile strength, modulus and rupture deformation were tested using an Instron TM 1122 (Norwood, Massachusetts, USA) universal testing machine. The tensile tests were performed with a 5 kN load cell at a test speed of 2 mm/min according to ASTM D790. An MFA2 LBG (Bergamo, Italy) extensometer was used for a more precise determination of the deformation of the materials. All trials were performed 10 times and the results shown are the average values obtained.

The polarity of corn stover fibers and bio-polyethylene was characterized by colloidal titration with methyl glycol chitosan (MGCh). The cationic demand was calculated using the colloidal titration technique developed by Terayama [37]. This technique consists of adding four milliliters of MGCh to 25 mL of a 1% fiber suspension. The suspension is kept in magnetic agitation for 1 min and centrifuged at 3000 rpm for 15 min. Then, 10 mL of the previous suspension is titrated with polyvinyl sulfate of potassium, using *O*-Toluidine blue as an indicator.

## 3. Results and Discussion

### 3.1. TMP Fiber Assessment

The main objective of the thermomechanical treatment was to extract the fibers from the stover increasing the percentage of cellulose and decreasing lignin content. More cellulose minds increasing the amount of hydroxyl groups prone to create bonds with the coupling agent. There are harder treatments, like chemo-thermomechanical or chemical treatments that further increase the percentage of cellulose, but show lesser yields. The yield of the used thermomechanical treatment is around 94% relative to the initial biomass. Harsher treatments can decrease this yield down to 65%. Other studies showed the advantages of a thermomechanical treatment in front of the other [16]. In this sense, Table 1 shows the variation in polarity of the corn stover fibers obtained through different treatments as well as the treatment yield.

The results showed that by increasing the severity of the treatment, the polarity of the fibers is reduced. However, these treatments reduce significantly the yield. While the fibers obtained by mechanical treatment present an absorption of 29.33 μeq/g, the chemical fibers only absorbed 10.11 μeq/g. On the other hand, the thermo-mechanical fibers absorbed 22.97 μeq/g, while only 3.95 μeq/g of MGCh were absorbed by biopolyethylene. This different polarity indicates that there are no strong chemical interactions at the fiber-matrix interface for composites without coupling agent [34].

The treatment also reduced or almost eliminated the percentages of ashes and extractives. The presence of extractives such as waxes that can degrade during compounding is positive because its degradation can cause decreases in the flowability of the materials [38].

Table 2 shows the chemical composition of untreated and treated corn stover fibers.

As shown, TMP fibers presented lower content of extractives, ash, lignin and hemicellulose with an increase of around 10% of the cellulose content.

### 3.2. Analysis of the Melt Flow Index

**Figure 2:**
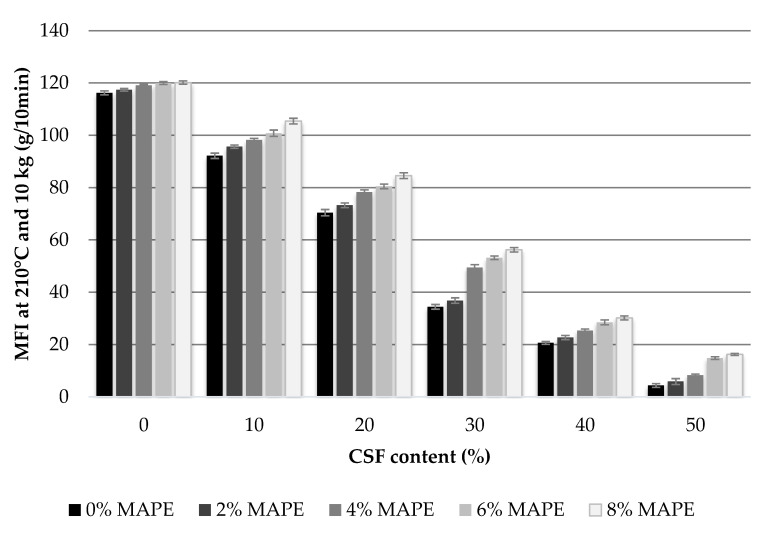
Melt Flow Index of Bio-polyethylene (BioPE) and Bio-polyethylene with corn stover fibers (BioPE/CSF) coupled and uncoupled composites.

In order to measure the effect of polyethylene maleic anhydride (MAPE) and corn stover fibers (CSF) on the flowability of the matrix and the composite materials, the MFI of the BioPE matrix and the CSF reinforced composites were measured. Figure 2 shows the results obtained for materials with MAPE contents ranging from 0 to 8 wt.% and CSF contents ranging from 0 to 50 wt.%.

The flowability of a fiber reinforced composite is mainly affected by the volume fraction of the reinforcement, the morphology of the fibers and its ductility [38]. CSF fibers, like other natural fibers are expected to show a ductile behavior, better than that of more brittle materials like glass fibers [39]. As shown, the effect of the fiber content on the MFI of the composites is noticeable. The flowability of the materials decrease with CSF content. This is related with the anchoring of CSF with the matrix, hindering the flow of the BioPE melt. The strong decreases of the MFI against CSF content indicates a ductile nature of the obtained fibers, as more brittle materials like glass fiber show less noticeable decreases of the MFI against its content [38,40]. Thus, the presence of CSF clearly affected the flowability of the composite materials, despite the presence or not of coupling agents. Similar behaviors of the MFI were observed for other natural fiber reinforced composites [25]. Despite this change on the flowability of the material the injection molding process allows adjustments on the pressure that permit the processing of all the materials.

The effect of the coupling agent was the opposite, leading to an increase of MFI of the composite materials with MAPE content. The MFI of the uncoupled 50 wt.% CSF reinforced BioPE was only 3.7%, the value of the matrix, and the same composite coupled with 8 wt.% of MAPE showed an MFI of 13.5% for the coupled matrix. This can be explained by the increasing number of interactions between the matrix and the CSF in the interphase. In fact, this stronger interphase increases the attrition phenomena during mixing, affecting the mean length of the reinforcements. Thus, the mean fiber lengths inside an uncoupled composite are expected to be shorter than those inside a coupled material. Short fibers are more easily oriented against flowing than long fibers. Thus, the increase of the MFI against MAPE contents indicates the creation of increasingly strong interphases and lower decrease of the length of the reinforcements. Similar results were observed with Jute reinforced PP materials [25,41].

### 3.3. Effect of MAPE and CSF Contents on the Tensile Strength of the Composites

**Figure 3:**
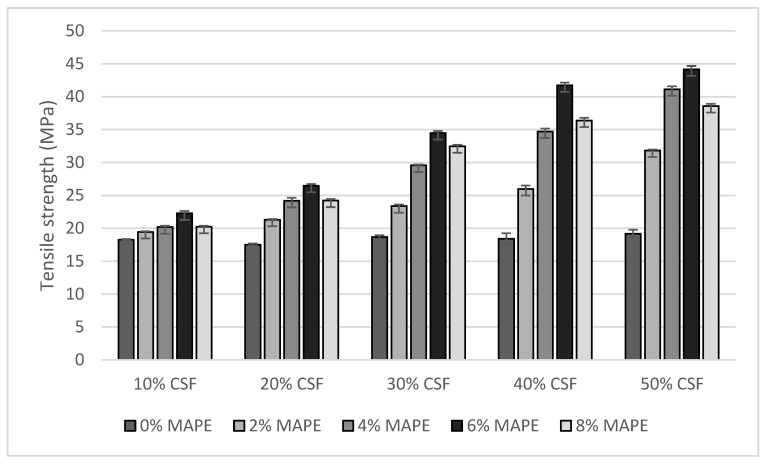
Evolution of the tensile strength of the composites against MAPE and CSF contents.

Figure 3 shows the evolution of the tensile strength of the composites against CSF and MAPE contents.

**Figure 4:**
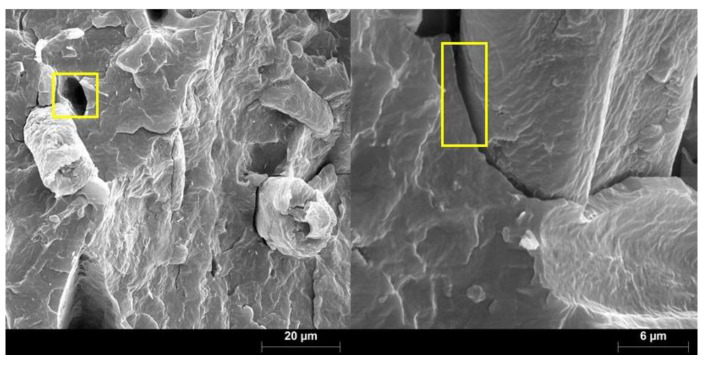
SEM micrographs of BiopPE reinforced with CSF.

In the absence of coupling agent all the composite materials showed similar tensile strength. In these composites CSF acted as filler instead of reinforcement since tensile strength values were similar to the one of the matrix (18.05 ± 0.15 MPa). The maximum strength was obtained for the composite with 50 wt.% CSF content, with a value 6% higher than the tensile strength of the matrix. Nonetheless, at a 95% confidence rate, the differences between the tensile strength of the uncoupled composites showed no statistical significance. This indicates a weak fiber-matrix interphase or the absence of such interphase. Most provably, CSF is poorly wetted by BioPE, showing the interphase noticeable voids all around (Figure 4). This hinders the transfer of loads from the matrix to the reinforcements, being been previously observed for similar reinforcements [42].

The presence of MAPE affected noticeably the tensile strength of the composites. This effect was most notable when the percentage of CSF increased. In all the cases the tensile strength of the composites increased with the presence of the coupling agent up to 6 wt.% MAPE content. Further MAPE content returned decreases of the tensile strength of the composites. The role of the coupling agent can be explained by the one hand providing ester bonds with CSF by chemical reactions between the maleic anhydride part of the coupling agent and the hydroxyl groups on the surface of the cellulosic fibers. On the other hand, the PE constituent of the coupling agent diffuses correctly and mixes with the matrix [43]. These two facts allow the creation of strong interphases that ensure a full wetting of the fibers and chemical interactions. This happens up to a certain content of coupling agent. For higher content than 6 wt.% such coupling agent tends to self-react and self-entangle, impacting negatively the strength of the interphase. This was observed for the composites with 8 wt.% MAPE content, that decreased its tensile strength in comparison with the materials with 6 wt.% of MAPE. This is a behavior previously reported in the literature for other natural fiber reinforced composites. Other natural fibers reinforced polyolefin composites showed that coupling agent contents in the range from 4 to 6 wt.% returned the highest tensile strengths.

CSF content had a very noticeable effect on the tensile strength of coupled composites, mainly for 6 wt.% MAPE coupled composites. These composites presented an increase of the tensile strength with respect to the matrix of 23.5, 46.7, 91.1 and 131.2% for 10, 20, 30 and 40 wt.% CSF contents, respectively. These results indicate the presence of an interphase and the good dispersion of the reinforcements inside the composite [44].

**Figure 5:**
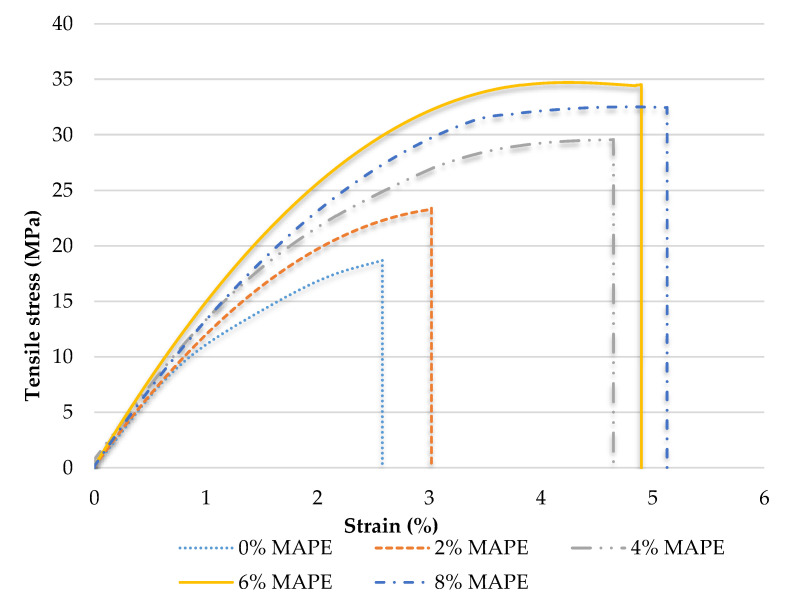
Stress-Strain curves for the 30 wt.% CSF reinforced BioPE composites at 0 to 8 wt.% MAPE contents.

Figure 5 shows the stress-strain curve for the uncoupled and coupled 30 wt.% of CSF composites with different MAPE content.

As shown, increasing coupling agent content led to an increasing of the tensile strength and strain at break of the composites. It must be noted how the tangent angle of the curves in the origin is very similar, revealing possibly similar Young’s moduli for these materials. The literature supports a limited effect of the strength of the interphase on the Young’s modulus of a material [3,17], thus there are similarities on the tangent angle. It is accepted that composite materials will fail due to the weakness of its phases: the matrix, the reinforcement or the interphase [42]. Usually the weakest phase is the interphase, which allows the fiber-matrix load transfer. This load transmission is done by transferring the matrix tensile loads to interphase shear loads and then again to tensile loads in the reinforcement section [45]. The stronger the interphase, the most capable will be such interphase to transfer loads without breaking and causing fiber slippage. If the interphase and the reinforcements are strong and long enough, respectively, the load transfer will end in reinforcement break instead of interphase failure [42]. In this sense, the aforementioned Figure 5 shows a progressive reinforcement of the interphase up to a 6 wt.% MAPE content.

In order to assess the strength of the interphase and the effect of the morphology of the fibers on the tensile strength of the composites, morphological and micromechanics analysis are proposed.

### 3.4. Analysis of the Fiber’s Morphology

It is known that the morphology of the reinforcement changes during the composite processing (mixing and injection molding in this study). The changes are mainly due to attrition phenomena that tends to decrease the average length of the reinforcement. On the other hand, the lumen of the fibers can collapse due to pressure leading also a change on the mean width of the reinforcement.

**Figure 6:**
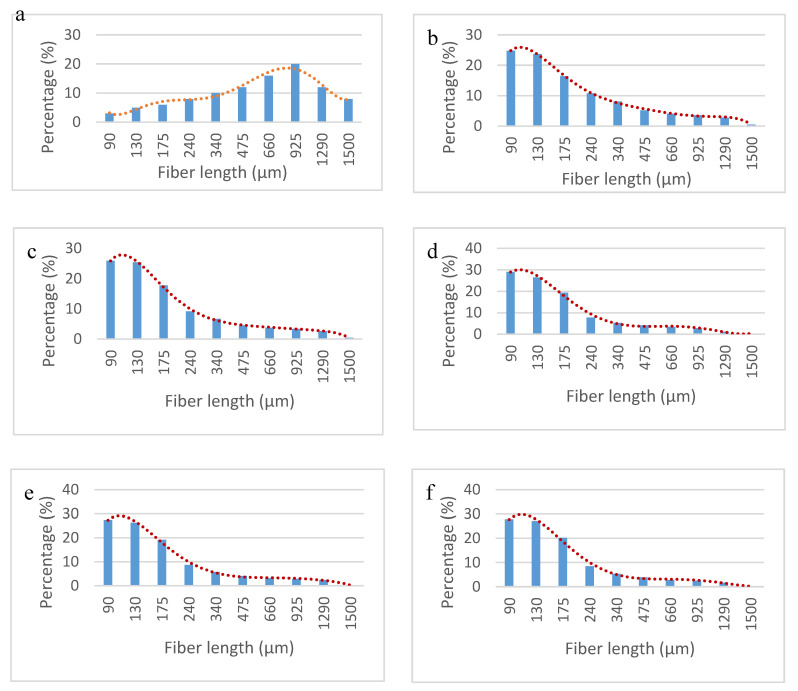
Fiber length distributions for the CSF; (**a**): Initial fibers, (**b**): 10 wt.% CSF, (**c**): 20 wt.%, (**d**): 30 wt.%: (**e**) 40 wt.%, (**f**): 50 wt.%.

Figure 6 shows the fiber length distributions obtained after extracting the reinforcements from the composites adding a 6 wt.% of MAPE.

The figure shows significant differences between the fiber’s length distribution before and after submitting the fibers to the mixing and mold injection processes. Initial fibers showed a mean arithmetic length of 695 µm. The fibers extracted from the composites ranging CSF content from 10 to 50 wt.% had a mean length of 264, 253, 220, 233 and 218 µm. The mean length decreased with CSF content. The decrease of the mean length clearly shows the effect of the attrition phenomena. This is in agreement with the analysis of the MFI.

Concerning to the mean fiber’s width, it was found only a slightly reduction from one batch to the other. The mean fiber width of the reinforcements after extracted from the composites was 17.2 ±0.2 µm. The untreated fibers showed a width of 21.3 µm. As stated previously, the most probable cause of this reduction is a lumen collapse [46].

### 3.5. Micromechanics of the Tensile Strength

As it was shown in Figure 3, the tensile strength of the composites evolved linearly with the reinforcement content allowing the use of linear models. A modified rule of mixtures for the tensile strength of short fibers semi-aligned reinforced composites can be formulated as [39]:

(1) $$\sigma_{t}^{C}=f_{c}\sigma_{t}^{F}V^{F}+\left(1-V^{F}\right)\sigma_{t}^{M*}$$

where $\sigma_{t}^{C}$ is the tensile strength of the composite, $\sigma_{t}^{F}$ the intrinsic tensile strength of the reinforcement and $\sigma_{t}^{M*}$ the contribution of the matrix or the tensile status of the matrix that corresponds with the strain at break of the composite. A coupling factor (f ) is used to equalize the contribution of the reinforcements due to fiber morphology and orientation, and interphase strength. The reinforcement volume fraction is represented by $V^{F}$. Except for the intrinsic tensile strength and the coupling factor, the rest of the parameters were experimentally obtained during the tensile tests of the matrix and the polymers. Thus, the equation shows two unknowns and cannot be solved. Therefore, the use of the Kelly and Tyson modified equation [47], with the solution provided by Bowyer and Bader [48], was proposed to obtain the intrinsic tensile strength of the reinforcement. Modified Kelly and Tyson equation is as follows [45]:

(2) $$\sigma_{t}^{C}=\chi_{1}\left(\overset{i=0}{\underset{Lc}{\sum }}\left[\frac{\tau \cdot l_{i}^{F}{\cdot V}_{i}^{F}}{d^{F}}\right]+\overset{j=Lc}{\underset{\infty }{\sum }}\left[\sigma_{j}^{F}{\cdot V}_{j}^{F}\cdot \left(1-\frac{\sigma_{t}^{F}{\cdot d}^{F}}{{4\cdot \tau \cdot l}_{j}^{F}}\right)\right]\right)+\left(1-V^{F}\right){\cdot \sigma }_{t}^{M*}$$

This equation divides the reinforcing fibers between sub-critical fibers (*l*_i_) and super-critical fibers (*l*_j_), depending on its length. Critical fibers are able to transmit thorough its surface enough load to break the fiber. The equation introduces an orientation factor ($\chi_{1}$) that accounts for the mean orientation of the fibers against the loads. The interfacial shear strength (*τ*) evaluates the strength of the interphase by measuring the shear strength that can be conveyed from the matrix to the fibers. This equation presents three unknowns, $\chi_{1}$, *τ* and $\sigma_{t}^{F}$. Nonetheless, Bowyer and Bader proposed a methodology that allowed finding the values of the interfacial strength and the orientation factor, leaving the intrinsic tensile strength of the fibers as remaining unknown. Although $\chi_{1}$ and τ usually are not known, values for these factors can be obtained from the stress-strain curve of the composite material by selecting two stress values (*σ*_1_ and *σ*_2_) at two different strains (*ε*_1_ and *ε*_2_). Bowyer and Bader calculated the contribution of the matrix (*Z*) from the determination of the elastic modulus of the matrix. The *Z* value is used to calculate the ratio *R* of the fiber contribution for the two strains studied. The equation with a proposed value of τ is used to calculate the ratio R*, the theoretical *R*. At this point the *R* and R* relationships are independent of o. Adjusting then the value of until R* = *R*, and this value is used in equation 2 to obtain the value of $\chi_{1}$, which is assumed to be the same for the two deformation values.

(3) $$R=\frac{\sigma_{1}-Z_{1}}{\sigma_{2}-Z_{2}}\;R^{*}=\frac{X_{1}+Y_{1}}{X_{2}+Y_{2}}$$

Once the parameters *X*_1_ and *τ* are known, using Equation (2) and taking into account that, *Lc* is equal to the intrinsic strength of the fiber by the average diameter of the fibers divided by two times tau, it is possible to determine the intrinsic resistance of the fibers.

Equation (2) was solved for the 30 wt.% CSF reinforced BioPE composites. Table 3 shows the experimental values used to solve such equation.

The contribution of the BioPE matrix was obtained from the experimental stress-strain curves of the polymer. Bowyer and Bader methodology uses the intrinsic Young’s modulus of the reinforcements. This value was computed by using Hirsch’s equation [49]:

(4) $$E_{t}^{C}=\beta \cdot \left(E_{t}^{F}\cdot V^{F}+E_{t}^{M}\cdot \left(1-V^{F}\right)\right)+\left(1-\beta \right)\frac{E_{t}^{F}\cdot E_{t}^{M}}{E_{t}^{M}\cdot V^{F}+E_{t}^{F}\cdot \left(1-V^{F}\right)}$$

where $E_{t}^{C}$, $E_{t}^{M}$ and $E_{t}^{F}$ are the Young’s modulus of the composite and the matrix and the intrinsic Young’s of the reinforcement, respectively. The parameter *β*, with a value of 0.4 balances the contribution of the fibers acting as parallel or perpendicular to the load. The development of Hisrch’s equation using Bowyer and Bader solution has reported values of the intrinsic modulus of the fibers around 16.4 GPa. Achieving the maximum value by the addition of 6% coupling agent. These values are in accordance with those previously obtained for this type of fibers [50].

Table 4 shows the obtained interfacial shear strength, orientation factor and intrinsic tensile strength for the 30 wt.% CSF reinforced BioPE composites.

The literature indicates that a strong interphase shows values in the range defined by Tresca ($\sigma_{t}^{C}$⁄2) and von Mises ($\sigma_{t}^{C}$⁄√3) criteria, thus, between 9.0 and 10.4 MPa [42,45]. All the coupled composites are inside this range proving the ability of MAPE for increasing the interphase interactions. The interfacial shear strength of the uncoupled composite is well below the mentioned values. Possibly the strength of this interphase is due to mechanical anchoring and friction phenomena. The 6 wt.% MAPE composite showed the highest interfacial shear strength. This was expected as this composite showed the highest tensile strength. Only 0.16 MPa separates the obtained value from the upper bound defined by von Mises criteria. Thus, the interphase can be labeled as strong.

The orientation factors can be used as verification parameter. The orientation is mainly defined by the geometry of the injection mold and the parameters used during the production of the specimens. Thus, this value changes slightly. Previous analysis showed that the value of the orientation factor ranged from 0.25 to 0.35, with a mean value of 0.3 [51,52,53]. All the obtained values are inside the mentioned range and thus considered correct. The orientation factors can be converted to a mean orientation angles (*α*) by the relation: $\chi_{1}=cos^{4}\left(\alpha \right)$ [45]. Then, the mean orientation angle of the composites adding 0 to 8 wt.% of MAPE are 43.4°, 43.4°, 42.4°, 42.3° and 42.7°, respectively. The obtained mean orientation was in agreement with previously reported results [16,42,45].

Although the reinforcing fibers used for all the composite materials were the same, the intrinsic tensile strength of the reinforcement changed with MAPE content (from 343.2 to 633.6 MPa). Obtaining different intrinsic tensile strengths can seem contradictory. Nonetheless, these values must be analyzed as the yield obtained from the strengthening capabilities of the CSF. Then, the higher the interfacial shear strength, the higher the yield. A bad interphase hinders a full exploitation of such reinforcing capabilities. Moreover, some researchers warn of the differences between intrinsic properties obtained by experimental or by micromechanics methods. In any case, the highest value shows an almost full exploitation of the fiber. The literature shows values between 460 and 670 MPa for CSF as polypropylene reinforcement, for uncoupled composite and coupled one respectively [15]. Thus, the obtained values were validated.

The 633.6 MPa value was used with equation 1 to evaluate the coupling factors of the composites. The obtained values for the composites adding 0, 2, 4, 6 and 8 wt.% of MAPE were 0.07, 0.09, 0.13, 0.16 and 0.14, respectively. The literature shows that coupling factors between 0.18 and 0.2 are optimum values. With these values the intrinsic tensile strength of CSF is between 505.4 and 561.6 MPa.

Table 5 shows the experimental values obtained for the 6 wt.% MAPE coupled composites against CSF content. These composites showed the highest tensile strengths and consequently took advantage of the strengthening capabilities of the reinforcement.

**Figure 7:**
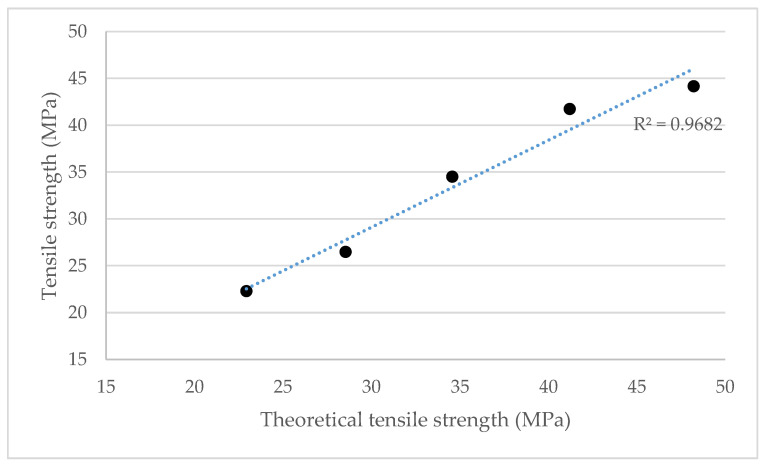
Correlation between experimental and theoretical tensile strengths of CSF reinforced BioPE composites.

As was shown in Figure 3, the tensile strength of these composites evolved linearly with CSF content. In order to assess the validity of the intrinsic tensile strength of CSF, this value was used with the modified rule of mixtures (Equation (1)) to evaluate a theoretic tensile strength of the composites. Figure 7 shows the obtained results.

The values were computed using a 0.16 coupling factor value. The obtained values for the 10 to 50 wt.% reinforced composites were 22.9, 28.5, 34.6, 41.2 and 48.2 MPa, respectively. Trend lines show a good correlation between experimental and calculated values. Nonetheless, the value corresponding with the composite reinforced with 50 wt.% of CSF showed the highest relative error (9.2%). A trend line that leaves out this value increases the correlation from 0.97 to 0.99 between the theoretical and experimental tensile strength values of the different compounds. Most provably the composite with the highest CSF contents showed a tensile strength lower than the potential due to a more difficult dispersion of the fibers. This is common for natural fiber reinforced composites and has been reported previously in the literature, where composites with reinforcement percentages equal to or higher than 50 wt.% tend to show lower tensile strengths than expected.

## 4. Conclusions

Corns stover fibers presented lower content of extractives, ash, lignin and hemicellulose with an increase of around 10% of the cellulose content than the untreated fibers.

The absence of maleated coupling agent led to composites with a weak or almost absence of fiber-matrix interphase. CSF was poorly wetted by the BioPE matrix showing the interphase noticeable voids all around.

The presence of MAPE allowed the creation of a good interphase and well dispersion of CSF. This led to an increase of the tensile strength and strain a break of the composites, being this effect more noticeable with CSF and MAPE content up to 6 wt.% (with a maximum increase of the tensile strength with respect to the matrix of 131.2% for 40 wt.% CSF content). Due to mechanical anchoring and friction phenomena, in conjunction with the creation of chemical bonds between the coupling agent and the fibers, a strong interphase with values in the range defined by Tresca and von Mises criteria was found for the coupled composites.

Fiber’s length distribution decreased after submitting the fibers to the mixing and mold injection processes, and this decrease was more noticeable for the composites with CSF content. This decrease was related to an attrition phenomenon, being in agreement with changes on the MFI.

## Figures and Tables

**Table 1:** Polarity and treatment yield of corn stover fibers and BioPE.

Raw Material	Treatment	Polarity (μeq/g)	Yield (%)
Corn Stover	Mechanical (MP)	29.33	99.1
	Thermo-mechanical (TMP)	22.97	93.8
	Chemical-thermo-mechanical (CTMP)	19.1	65.6
	Chemical pulp (CP)	10.11	51.45
Bio-Polyethylene	-	3.95	-

**Table 2:** Chemical composition of the surface of untreated and thermo-mechanical digestion process (TMP) corn stover.

	Ash (%)	Extractives (%)	Ligni (%)	Hemicellulose (%)	Cellulose (%)	Length * (µm)	Diameter (µm)
Corn Stover	3.15	3.08	15.75	25.40	52.62	-	-
TMP Corn Stover fibers	0.67	0.94	13.24	23.67	61.48	695	21.3

* Average of length weighted in length.

**Table 3:** Tensile strength, Young’s modulus and strain at break and contribution of the matrix for the 30 wt.% CSF reinforced BioPE composites.

MAPE (%)	V^F^	$\sigma_{t}^{C}$ (MPa)	$E_{t}^{C}$ (GPa)	$\varepsilon_{max}$ (%)	$\sigma_{t}^{m^{*}}$ (MPa)
0	0.219	18.68 ± 0.25	2.36 ± 0.03	2.58 ± 0.82	12.13
2		23.37 ± 0.25	2.39 ± 0.09	3.02 ± 0.13	13.09
4		29.56 ± 0.10	2.53 ± 0.01	4.65 ± 0.13	15.55
6		34.50 ± 0.28	2.92 ± 0.06	4.90 ± 0.07	15.83
8		32.46 ± 0.24	2.63 ± 0.02	5.13 ± 0.082	16.07

**Table 4:** Micromechanic parameters of the 30 wt.% CSF reinforced BioPE composites obtained by using Kelly and Tyson modified equation.

MAPE (%)	*τ* (MPa)	*χ*_1_ [0-1]	$\sigma_{t}^{F}$ (MPa)
0%	6.65	0.280	343.18
2%	9.47	0.280	396.56
4%	9.53	0.297	524.50
6%	10.26	0.300	633.56
8%	9.85	0.293	567.54

**Table 5:** Tensile strength and strain at break and contribution of the matrix of the 0 to 50 wt.% CSF reinforced BioPE composites coupled with 6 wt.% of MAPE.

CSF (%)	V^f^	$\sigma_{t}^{C}$ (MPa)	$\varepsilon_{max}$ (%)	$\sigma_{t}^{m^{*}}$ (MPa)
0	0	18.05 ± 0.15	10.59 ± 0.53	-
10	0.068	22.28 ± 0.33	6.44 ± 0.16	17.21
20	0.141	26.48 ± 0.25	5.66 ± 0.12	16.58
30	0.219	34.50 ± 0.28	4.90 ± 0.07	15.83
40	0.304	41.73 ± 0.41	4.15 ± 0.08	14.93
50	0.395	44.17 ± 0.50	3.24 ± 0.21	13.51
